# Manure amendment increases the content of nanomineral allophane in an acid arable soil

**DOI:** 10.1038/s41598-017-14445-2

**Published:** 2017-10-27

**Authors:** Jianchao Zhang, Jian Xiao, Siliang Li, Wei Ran

**Affiliations:** 1 Institute of the Surface-Earth System Science Research, Tianjin University, Tianjin, 300072 China; 2 Jiangsu Provincial Coordinated Research Center for Organic Solid Waste Utilization, Nanjing Agricultural University, Nanjing, 210095 China

## Abstract

Natural nanoparticles are of central importance in the environment, e.g. sorption of soil organic carbon (SOC) and contaminants. A large number of study have focused on the metal binding, transport and ecotoxicity of nanoparticles. Fertilizer amendments are routinely applied to arable soils and induce changes in soil chemical, physical and biological properties. However, the effects of fertilizer amendments on natural nanoparticles are still unknown. In this study, soil nanoparticles were separated from acid red soil (Ferralic Cambisol) including long-term (26 years) treatments of unfertilized control (CK), chemical nitrogen, phosphorus and potassium fertilizers (NPK) and raw pig manure (M). The results from high-resolution transmission electron microscopy (HRTEM) and Fourier-transformed infrared (FTIR) spectroscopy indicated that nanoparticles in red soil were heterogeneous organo-mineral associations with irregular shapes, regardless of fertilization history. In addition, kaolinite and allophane occurred in the soil nanoparticles. Intriguingly, we found the content of allophane under M treatment (0.64 g kg^−1^) was much higher than under CK and NPK treatments. However, the CK (0.27 g kg^−1^) and NPK (0.21 g kg^−1^) had similar allophane concentrations. Our study may indicate long-term organic manure amendment initializes positive feedback loop for further SOC sequestration. However, the mechanisms for the enhancement of nanomineral allophane by manure amendment deserve further investigation.

## Introduction

Soil particles are traditionally classified into sand, silt and clay fractions according to their sizes^1,2^. Recently, with the rapid development of nanotechnology and nanoscience, natural nanoparticles are of increasing interest and concern. The nanoparticles may display unique physical properties and chemical behaviors because of their high surface area to volume ratio and/or quantum effects. It has been suggested that nanoparticles could be highly effective in soil organic carbon (SOC) sequestration^3^.

Soils are complex ecosystems with diverse compositions, which generally include particles of nanomolecular size^4^. Under the effect of either biotic or abiotic processes, all the minerals go through a nanophase stage during formation^5,6^. Natural nanoparticles could occur as nanominerals (which are defined as minerals such as allophane that only exist in the nano-size range (<100 nm), or clays that only exist with at least one dimension in that size rage) or as mineral nanoparticles (which are defined as minerals that can also exist in larger sizes, e.g., most known minerals)^5^. A typical example of a nanomineral is allophane, which is ubiquitous in soil environments^6,7^. Allophane is amorphous nanomineral with an aluminum/silicon (Al/Si) ratio that may vary between 1 and 2. Irrespective of origin and chemical composition, the unit particle of allophane is a hollow spherule with an outer diameter of 3.5–5.0 nm and a wall thickness of 0.7–1.0 nm^4^. The spherules form clusters which combine to form larger aggregates that can be up to 60–100 nm in diameter^8^. The nanoparticles control or dramatically affect the soil physical and chemical properties because of their large surface area, large number of surface functional groups per unit of mass and unique electrical or magnetic properties^9,10^. For example, allophane could retain up to 12% of SOC^3^. The stability of organic C in Acrisols and Andosols has been ascribed to their strong and intimate association with allophane^11,12^.

A wide variety of variables, such as soil pH, organic carbon content and dissolved organic matter concentration, in addition to the presence of impurities may affect the formation and phase transformation of natural nanoparticles^13–16^. Fertilization practices are routinely applied to maintain or improve the crop yields of arable soils. In addition, fertilization practices can also induce changes in soil chemical, physical and biological properties^17^. Organic manure has become the recommended management practice in the acid soils of China^18^. Many studies have revealed that manure application not only increases soil pH and SOC concentration but also decreases the exchangeable Al and other toxic Al species in acid soils^19–21^. Nevertheless, the effect of long-term fertilizer amendments on the nanoparticles in acid soils remain poorly understood. Thus, the objectives of this study were as follows: (1) to characterize the morphology, mineral phases and composition of soil nanoparticles in the acid soil; (2) to investigate the influence of manure amendment on the contents of nanomineral allophane. On the other hand, the field of nanoscience is of crucial importance to the soil sciences because many of the natural components of soils are nanoparticulate or contain nanoscale feature. The results will help further our understanding of the feed-back of long-term manure amendment in acid red soil.

## Results

### Morphology and composition of the soil nanoparticles

**Figure 1:**
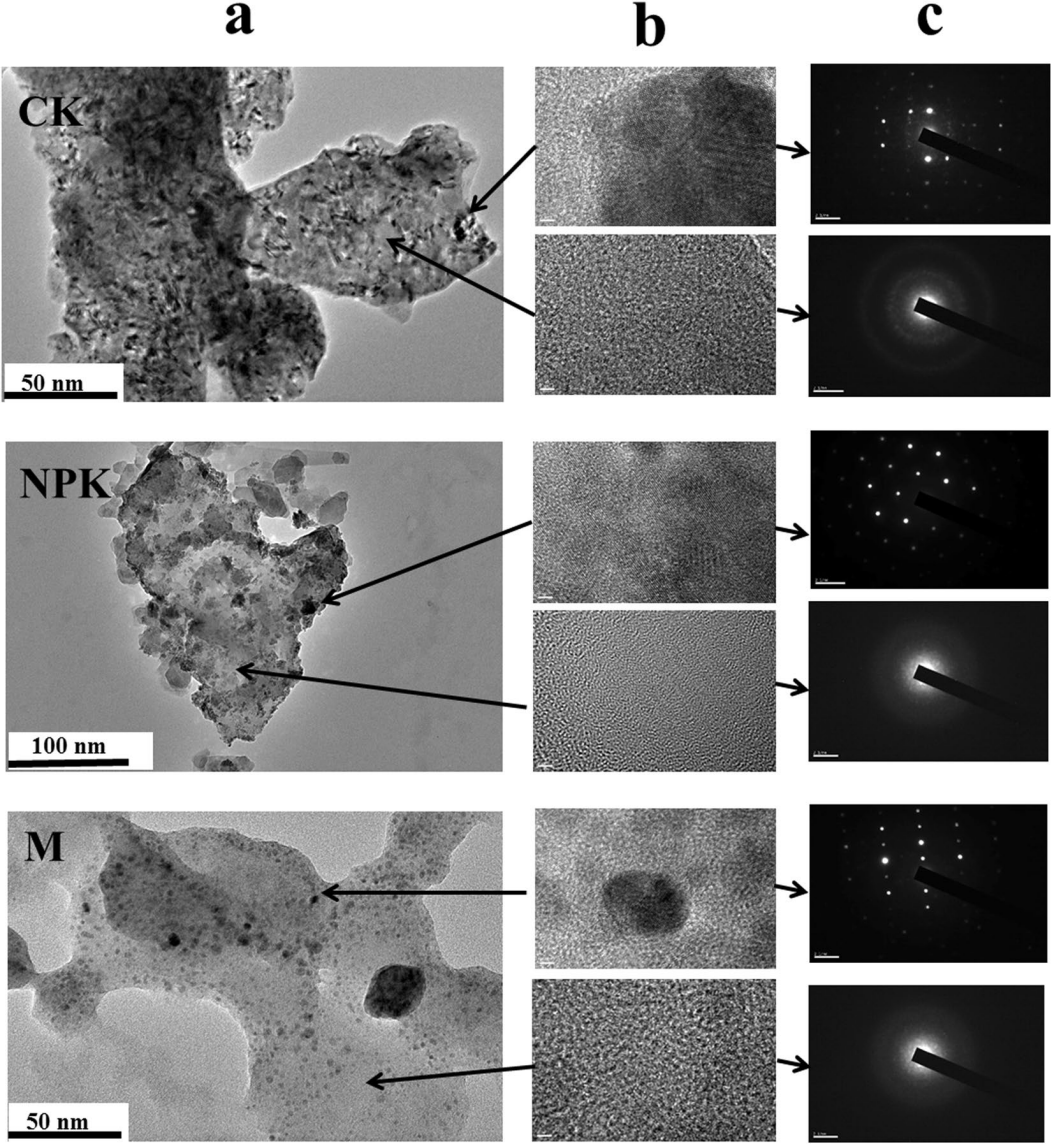
Transmission electron microscopy (TEM) of the soil nanoparticles from red soil under long-term fertilization. Column a: typical TEM images of the soil nanoparticles; Column b: high resolution TEM (HRTEM) images of the corresponding spot indicated by the arrow; Column c: selected area of electron diffraction (SAED) patterns of the corresponding region.

Soil nanoparticles from different fertilization treatments were observed by Transmission electron microscopy (TEM) at different scales (Fig. 1). TEM images showed that soil nanoparticles from different fertilization treatments all had irregular shapes (the sizes varied from less than 10 to 100 nm). In addition, the nanoparticles may be clustered and aggregated (approximately 50–250 nm).

**Figure 2:**
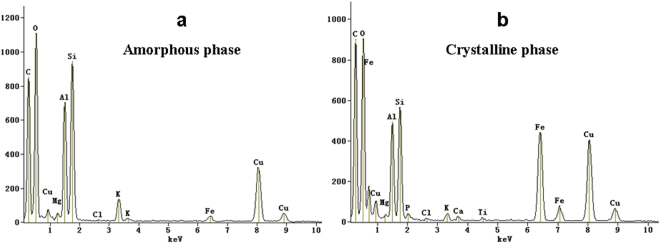
TEM-EDS spectrum of (**a**) light grey area (amorphous phase as indicated by arrow) and (**b**) black area (crystalline phase as indicated by arrow) in the TEM image of soil nanoparticles from M in Fig. 1. Copper (Cu) originate from the grid holder. TEM-EDS: TEM equipped with an energy dispersive X-ray spectrometer.

There were distinct light grey and black areas in the TEM images of soil nanoparticles among all the treatments. In addition, high resolution TEM (HRTEM) images and selected area of electron diffraction (SAED) patterns provide information about the structural characteristics and polycrystallinity in the two regions. Here, the clear lattice fringes of HRTEM images (column b of Fig. 1) and the typical spotty ring patterns of SAED patterns (column c of Fig. 1) were found in all black spots, which indicated that the nanoparticles appearing in black spots were well crystallized. The amorphous nanoparticles were dominated by C, O, Si and Al based on HRTEM-EDS analyses (Fig. 2a). These TEM results suggested that crystalline mineral in the nanoparticles consists mainly of Al- and Fe- (hydro-)oxides, and the amorphous mineral consists mainly of Al (hydro-)oxides. In addition, the data showed that the crystalline nanoparticles were dominated by C, O, Si, Fe and Al based on a number of HRTEM-EDS analyses (Fig. 2b). On the other hand, the absence of lattice fringes in HRTEM images and the absence of spots in SAED patterns (Fig. 1) revealed that the light grey areas in the nanoparticles were in an amorphous phase.

### FTIR spectroscopy of the soil nanoparticles

**Figure 3:**
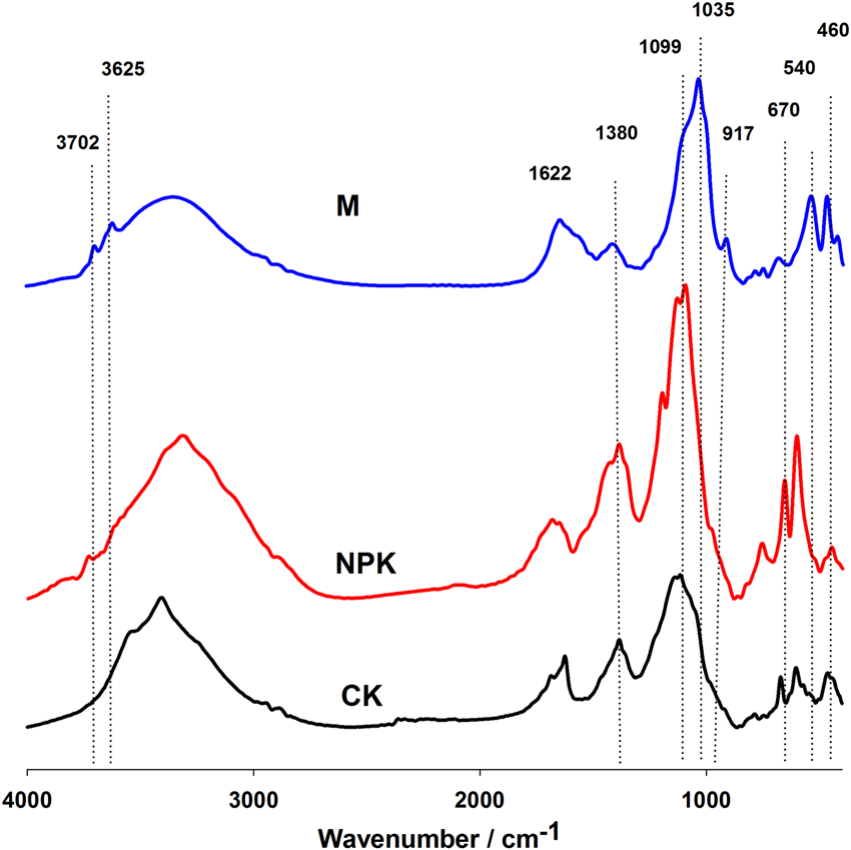
Fourier-transformed infrared (FTIR) spectra of the soil nanoparticles from red soil under long-term fertilization treatments.

Fourier-transformed infrared (FTIR) spectroscopy of the freeze-dried soil nanoparticles were shown in Fig. 3. The peaks near 2926, 1622 and 1380 cm^−1^ were assigned to aliphatic C-H stretching, aromatic C = C, and C-H bending of CH_2_ and CH_3_ groups of organic matter, respectively^22^. The three peaks (2926, 1622 and 1380 cm^−1^) could be found in all the treatments. The peaks at 670 and 460 cm^−1^ that originated from the peculiar wall structure of allophane ((HO)Si(OAl)_3_) were also found in all the treatments^23^. The absorption peaks at 3700, 3620 and 915 cm^−1^ that are attributed to the -OH group on kaolinite could be found in the nanoparticles^24^. In addition, the FTIR analyses demonstrated the varying composition of soil nanoparticles from different fertilization treatments. The absorption bands at ~1035, ~917 and ~540 cm^−1^ that are ascribed to proto-imogolite^25^ could only be found in M treatment.

### Contents of allophane and amorphous Al oxides

**Figure 4:**
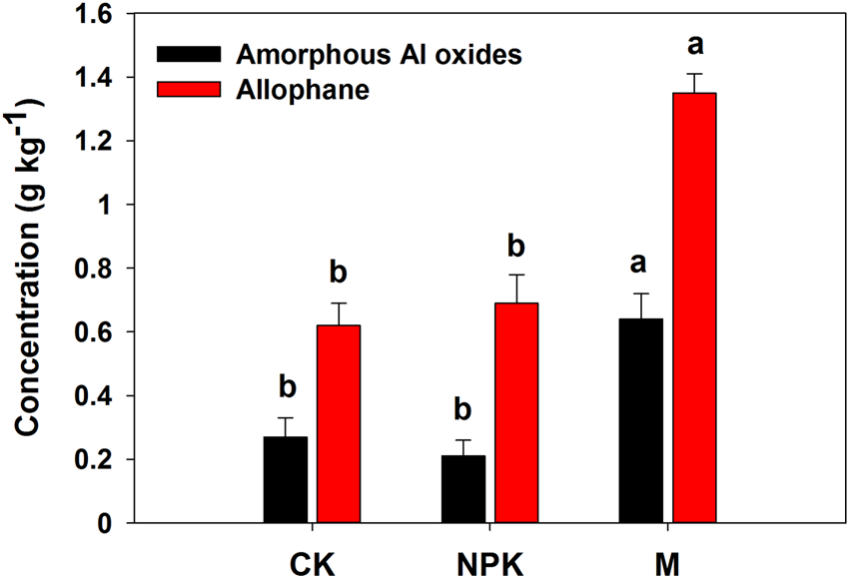
Effects of long-term organic manure amendment on the concentrations of nanomineral allophane and amorphous Al oxides. Fertilizer treatments with different lowercase letters are significantly at *P* < 0.05.

Allophane content in M treatment was higher than in CK and NPK treatments (Fig. 4). For allophane content in the present study, the treatments can be arranged in following order: M > CK ≈ NPK. Furthermore, contents of amorphous Al oxides were also changed after 26 years of manure amendment (Fig. 4). Amorphous Al oxides contents were lowest in CK treatment (0.62 g kg^−1^) and highest in M treatment (1.35 g kg^−1^). Contents of amorphous Al oxides can be arranged in the following order: M > NPK ≈ CK.

## Discussion

### The morphology and mineral composition of soil nanoparticles

The TEM images of the extracted soil nanoparticles indicated the particles had irregular shapes with at least one dimension less than 100 nm. The nanoparticles may be clustered and form aggregates in aqueous suspension. However, sonication segregates particles and allows nanoparticles to remain in the nanoscale size distribution.

Regardless of fertilization treatments, nanoparticles in red soil were heterogeneous organo-mineral associations, and consisted of mineral nanoparticles (i.e. kaolinite) and nanomineral (i.e. allophane) according to the FTIR spectra and TEM observations. The heavily weathered and leached red soil (Ferralic Cambisol) is characterized by low pH and SOC, and contains large amount of kaolinite clay minerals^26^. Our result showed that the kaolinite could also be found in the nanoscale. Additionally, allophane was also found in the acid red soil besides in volcanic ash soil^27^.

### Manure amendment increases the nanomineral allophane content

Nanoscale materials have chemical reactivity and physical properties that differ from those of corresponding bulk materials, and thus the nanosized particles are attracting attention. The nanoparticles can participate in essential ecological services that range from the regulation of SOC and water storage and element cycling to the transport and adsorption of contaminants^15^. The nano-sized mineral particles in soil were claimed to play an important role in SOC sequestration because of their large specific surface areas and high surface reactivity^28^. The sequestration of SOC in Acrisols and Andosols has been ascribed to their strong and intimate association with allophane^11^. The SOC stabilizing capacity of soil minerals decreases in the following order: allophane > smectite > illite > kaolinite^29^. Allophane is amorphous nanomineral with a hollow spherule structure, and it has a high cation/anion exchange capacity as well as extensive variable-charged surfaces^27^. Allophane was used as an adsorbent and its surface area as high as ~1000 m^2^ g^−1^, which is often larger than activated carbon^4,23^. The ligand exchange between the surface hydroxyls of allophane and the carboxyl groups of organic matter promote SOC stabilization^3^. Furthermore, allophane aggregates could prevent SOC from being accessed by microorganisms or their enzymes^11^. It is interesting to find that the allophane concentration in M treatment was higher than in CK and NPK treatments. The higher allophane concentration in M treatment may suggest M treatment had higher SOC binding and stabilizing capacity of soil than CK and NPK treatments.

Numerous studies have been conducted on the behavior and fate of engineered nanoparticles released into the environment, especially with the aim of examining their effects on the ecosystems and humans^16^. In contrast, the knowledge base on the formation and fate of naturally occurring metal nanoparticles in soil is sparse. All minerals go through a nano phase stage during formation under the effect of either biotic or abiotic processes^5,30^. The nanomineral could be form under biotic processes or nanobiomineralization. Two processes are designated in nanobiomineralization^16^ as (1) biologically controlled mineralization (BCM) and (2) biologically induced mineralization (BIM). The formation and growth of nanoparticles are entirely controlled by the organisms during BCM processes and the nanominerals are usually formed in the cells^16,31^. In contrast, nanoparticles were formed as a result of metabolic processes in the BIM process with no function being particularly controlled by microorganisms^16,32,33^. Biomineralization process in the formation of allophane in acid soil are still unknown. However, it is well known that long-term manure amendment could increase the soil biological activity compared with the NPK treatment^34,35^. In addition, in the biomineralization process, organic molecules are used as templates to control the size and shape of nanoparticles^30^. Thus, the long-term manure amendment would facilitate the formation of nanomineral allophane. Furthermore, some studies also showed that agricultural practices could also contribute to the formation of amorphous oxides or nanomineral in a relatively short time in volcanic ash soils^36,37^.

In addition, the soil pH significantly influences the formation and aggregation of nanomineral^38^. Allophane does not usually occur in soils unless the soil pH is higher than 4.7^39^. Large concentration of allophane could occur when soil pH ranges from 5.5 to 6.8^38^. The results from Garrido and Matus^40^ also suggested that high soil pH and silica gel could promote allophane formation. Thus, the high soil pH in M treatment (soil pH = 6.63) may facilitate the formation of allophane compared with CK (soil pH = 5.47) and NPK (soil pH = 4.15) treatments.

Nevertheless, the M and NPK treatments could both change the soil chemical properties (Table 1), microbial community and biological activity^35^ compared with CK. Typical Gram-negative bacteria biomarkers were 27% higher in manure treatment than in CK^41^. NPK treatment resulted in a 15% decrease in bacteria biomarkers compared with CK^41^. Labile organic matter, microbial biomass C and microbial metabolic activity (dehydrogenase activity per microbial biomass C) was significantly higher (p < 0.05) under manure treatment than under CK and NPK treatments^42^. However, in the current study, the nanomineral allophane was only increased by M treatment, and the NPK and CK had similar allophane concentration. In many cases, the formation of nanoparticles occurs via combination of various factors and processes^16^. Therefore, the increase of allophane by long-term manure amendment may be an integrated process. This study suggested more study in the future about the mechanisms of allphane enhancement in the acid red soil.

**Table 1:** Soil characteristics after 26 years of fertilization treatments in the acidic red soil.

Treatment	Corn grain yield (kg ha^−1^)	pH (H_2_O)	SOC^a^/g kg^−1^	Exchangeable H/cmol(+) kg^−1^	Exchangeable Al /cmol(+) kg^−1^	Base Saturation /%	EC^b^/μS cm^−1^	Clay (<2 μm) contents / %	Silt (2–20 μm) contents / %
CK	1064.32 (105.44) c	5.47 (0.21) b	8.63 (0.32) c	0.55 (0.04) a	0.08 (0.02) b	92.45 (1.45) a	42.34 (2.34) c	30.51 (0.32) a	47.33 (0.33) a
NPK	2756.44 (174.72) b	4.15 (0.24) c	10.65 (0.78) b	0.53 (0.07) a	5.57 (0.77) a	38.50 (2.65) b	137.56 (4.44) a	30.58 (0.12) a	45.43 (0.41) b
M	3652.54 (223.69) a	6.63 (0.28) a	16.33 (0.88) a	0.37 (0.05) b	0.04(0.01) b	97.00 (4.23) a	111.87 (7.81) b	29.05 (0.23) b	46.29 (0.53) ab

The data was showed as average value (n = 3). Standard deviations in parentheses. Results followed by different letters are significantly different at *P* < 0.05.

^a^SOC: Soil organic carbon.

^b^EC: Electrical conductivity.

CK, without fertilization; NPK, chemical NPK fertilizer; M, raw pig manure.

### Enhancement of amorphous Al oxides content by manure amendment

Al is abundant in acid soils and Al can precipitate as amorphous minerals, which are very reactive towards SOC^43^. Allophane is amorphous Al oxides. The present study showed allophane could be enhanced by the M treatment compared with CK and NPK, however the effect of manure amendment on the concentration of amorphous Al oxides is still unclear. In the TEM observations, the proportion of amorphous material (grey color) in the nanoparticles were relatively higher in M treatment than in CK and NPK treatments (Fig. 1). In addition, the quantitative assay demonstrated the interesting result that the long-term organic manure amendment increased the content of amorphous Al oxides in acid soil (Fig. 4). This is supported by the other studies that organic ligands can incorporate into the network structure of amorphous Al minerals and thus prevent the formation of crystalline Al minerals^44,45^. A recent microcosm experiment provided direct evidence that the organic acids from the plant roots or manure promoted the transformation of mineral from crystalline to of amorphous phases^46^. Abundant studies showed that long-term manure amendment could increase the soil organic acids concentrations^47^, thus increase the amorphous Al concentration.

### Implications for soil organic carbon stabilization

In mineral soils, the majority of compounds are intimately associated with reactive mineral phases, e.g. amorphous or nano-sized minerals bind organic matter through their large surface area and various bonding sites^11,48^. Abundant studies revealed that amorphous Al oxides and allophane play an important role in the SOC stabilization of Acrisols and Andosols^12^.

It is well known that long-term manure application could control the accumulation of SOC^49,50^. This study increased our knowledge into the feed-back of long-term manure amendment in acid red soil (Ferralic Cambisol). The red soil characterized by low pH (<5.0) and low SOC (<10 g kg^−1^). Results (Table 1) showed that long-term manure amendment increased the SOC concentration compared with the CK and NPK treatments. In addition, our study indicated that the long-term application of manure may enhance the allophane contents and nanomineral allophane. Thus, based on the benefit of amorphous Al oxides and nanomineral allophane in SOC stabilization, the continuous organic carbon amendment may initialize positive feedback loop by increasing the concentrations of amorphous Al oxides and nanomineral allophane for further SOC sequestration. Our findings provide a management practice for regulating mineral availability and formation of amorhphous Al oxides and nanomineral allophane in the arable soil, which will be beneficial for managing the global C cycle. Our study also challenges the long-standing conceptual view that the formation of amorphous minerals are very slow processes and cannot be detected in a relative short time.

## Materials and Methods

### Experimental site and sampling design

The surface (0–20 cm) soil samples were collected in June 2016 from a long-term field experiment, which was initiated in 1990 at the experimental station of the Chinese Academy of Agricultural Sciences, Qiyang (26°45′N, 111°52′E), Hunan Province, southern China. The soil type is red soil (Ferralic Cambisol based on World Soil Classification) that developed from Quaternary red clay. The site represents a typical agricultural region in the hilly land area of subtropical China. The red soil could be classified as clayey and contains large amount of Fe and Al oxides and kaolinite clay minerals.

The long-term experiment was comprised of treatment plots (20 m × 10 m) with three replicates in a randomized complete block design. The long-term fertilizer experiment has eight treatments, and three treatments were used in this study: (1) CK, unfertilized control; (2) NPK, chemical nitrogen, phosphorus and potassium fertilizers and (3) M, raw pig manure. Six randomly selected soil cores (approximately 5 cm in diameter) were taken from each plot and mixed to form a composite sample. All samples were air-dried and sieved through 2-mm sieve.

The cropping system for the field experiment was winter wheat (*Triticum Aestivum* L.)-summer corn (*Zea mays* L.) rotations. For each year, summer corn was sown between the wheat strips in early April and harvested in July; winter wheat was sown in early November and harvested in May in the following year. The straw and grain were removed after harvest.

For the treatments of NPK and M, the applied total amount of N (manure or urea) was the same for each year. For NPK, the N was applied as urea at 300 kg N ha^−1^ year^−1^, P as single superphosphate at 53 kg P ha^−1^ year^−1^, and K as KCl at 100 kg K ha^−1^ year^−1^, respectively. The raw pig manure was obtained from local farms annually (average water content: 70%; total organic C content: 36%; and total N content: 1.8%). For annual input, 30% of total N was applied when wheat was planted, and 70% of total N was applied when corn was planted. Both chemical fertilizers and manure were applied at depth of 10 cm, followed by sowing of each crop and then covering with surface soil.

### Soil chemical properties analysis

Soil pH was determined with a pH electrode in a soil to water ratio of 1:5 (Multiline F/SET-3, Germany). The SOC contents were measured with a CN analyzer (Vario EL, Germany). Electrical conductivity (EC) was measured in a 1:2.5 water extracted using the electrode conductivity meter (Multiline F/SET-3, Germany). Exchangeable acidity (H^+^  + Al^3+^) and exchangeable Al were determined by double titration and exchangeable H^+^ was calculated by the difference between the two^51^. Exchangeable cations (Ca^2+^, Mg^2+^, K^+^, Na^+^) were extracted with 1 mol L^−1^ NH_4_Cl determined by inductively coupled plasma-optical emission spectrometry (ICP-OES, Vista-Pro, Varian, USA). Cation exchange capacity (CEC) was calculated as the sum of exchangeable base cations (Ca^2+^, Mg^2+^, K^+^, Na^+^), exchangeable H^+^ and Al^3+^. Base saturation (BS) was estimated as the percent of CEC occupied by exchangeable base cations (Ca^2+^, Mg^2+^, K^+^ and Na^+^)^52^. Soil particle size distribution (clay (<2 μm), fine silt (2–20 μm) sand (20–2000 μm)) was determined using a Mastersizer 2000 laser grain-size analyser (Malvern, UK) with a range of 0.02 μm to 2000 μm^53^. Selected soil characteristics after 26 years of fertilization treatments are showed in Table 1. In addition, total N, soil acid buffering capacity after long-term fertilization treatments could be found in our previous study^18^.

### Extraction of soil nanoparticles

The extraction of soil nanoparticles was conducted as described by Li, *et al*.^54^. Briefly, 3 g of soil was ultrasonically dispersed in 80 mL of water in a 100 mL glass beaker by a probe-type ultrasonic vibrator, which operated with stable power with an output of 60 kJ energy and 80% amplitude. The glass beaker was placed in a cooling bath below 25 °C throughout the sonication to prevent the effects of high temperature from affecting the stability of soil organic colloids. After sonication, the aqueous suspension was passed through a 50 μm sieve; the sieved material was suspended and placed in a 50 mL centrifuge tube and centrifuged at 3500 g for 24 min and the supernatant was collected. The material was centrifuged to obtain the desired particle size of less than 100 nm based on Stokes’ Law^55^.

### Transmission electron microscopy (TEM) measurements

TEM was used to determine the physical size and chemical composition of the nanoparticles in aqueous suspensions. A drop of the soil nanoparticles suspension was evaporated on a copper grid. The morphology of the soil nanoparticles was observed by TEM (JEOL, Tokyo, Japan) operated at an accelerating voltage of 200 kV. The microscope was equipped with an energy dispersive X-ray spectrometer (EDS). Cu could not be measured as the grids are made of copper and all spectra therefore contain a Cu peak. The structural characterization of the nanoparticles was conducted using TEM with high-resolution transmission electron microscope (HRTEM) images, EDS, and selected area electron diffraction (SAED) analysis. The regions for the HRTEM and SAED analysis were randomly chosen for all the treatments and three spots for each treatment were selected.

### Fourier-transformed infrared (FTIR) spectroscopy measurements

The samples of soil nanoparticles were freeze-dried before analysis. Samples were diluted in potassium bromide (KBr, IR grade) prior to analysis (2 mg of sample in 200 mg of KBr). KBr had been dried at 105 °C beforehand to minimize interference in FTIR spectra due to moisture. The FTIR spectra was obtained by collecting 200 scans with a Nicolet 370 FTIR spectrometer. The analysis was carried out in the mid-infrared region from 4000 cm^−1^ to 400 cm^−1^. Each spectrum was corrected against pure KBr and the ambient air as a background spectrum.

### Quantification of the allophane and amorphous Al in soil

Aluminum extracted with 0.2 mol L^−1^ solution of ammonium oxalate-oxalic acid (Al_o_) represents the sum of Al in organic and amorphous complexes. In addition, silicon was also extracted with 0.2 mol L^−1^ solution of ammonium oxalate-oxalic acid (Si_o_). The pool of Al extracted with 0.1 mol L^−1^ solution of sodium pyrophosphate (Al_p_) is considered to estimate the total organically bound Al. All extracts were filtered through 0.45 μm filters (Millipore Corporation, USA). The Al contents in the extracts were measured by inductively coupled plasma optical emission spectrometer (ICP-OES). The amorphous Al oxides were calculated from Al_o_-Al_p_^56^.

Allophane content was calculated using a formula^11^:

$$\begin{array}{c}{\rm{Allophane}}( \% )=100\times {{\rm{Si}}}_{{\rm{o}}}/[23.4-5.1({{\rm{Al}}}_{{\rm{o}}}-{{\rm{Al}}}_{{\rm{p}}})/{{\rm{Si}}}_{{\rm{o}}}]\\ {{\rm{Si}}}_{{\rm{o}}}:{\rm{Ammonium}}\,{\rm{oxalate}}\,{\rm{extractable}}\,{\rm{Si}}\end{array}$$

### Statistical analysis

Data represent arithmetic means and the standard deviation (SD) of three replicated analysis. One-way analysis of variance (ANOVA) followed by Duncan multiple comparison test was used to check for quantitative differences between treatments. *P* < 0.05 was considered to be statistically significant. Statistical analyses were performed using SPSS version 13.0.
